# Wallerian degeneration: gaining perspective on inflammatory events after peripheral nerve injury

**DOI:** 10.1186/1742-2094-8-110

**Published:** 2011-08-30

**Authors:** Andrew D Gaudet, Phillip G Popovich, Matt S Ramer

**Affiliations:** 1 Department of Neuroscience and Center for Brain and Spinal Cord Repair, College of Medicine, The Ohio State University, 770 Biomedical Research Tower, 460 West 12th Ave, Columbus, OH, 43210, USA; 2 International Collaboration On Repair Discoveries (ICORD), Vancouver Coastal Health Research Institute, and Department of Zoology, University of British Columbia, 818 West 10th Ave, Vancouver, BC, V5T 1M9, Canada

**Keywords:** Macrophage, microglia, axotomy, Wallerian degeneration, phagocytosis, neuroinflammation, inflammation, spinal cord injury, galectin-1

## Abstract

In this review, we first provide a brief historical perspective, discussing how peripheral nerve injury (PNI) may have caused World War I. We then consider the initiation, progression, and resolution of the cellular inflammatory response after PNI, before comparing the PNI inflammatory response with that induced by spinal cord injury (SCI).

In contrast with central nervous system (CNS) axons, those in the periphery have the remarkable ability to regenerate after injury. Nevertheless, peripheral nervous system (PNS) axon regrowth is hampered by nerve gaps created by injury. In addition, the growth-supportive milieu of PNS axons is not sustained over time, precluding long-distance regeneration. Therefore, studying PNI could be instructive for both improving PNS regeneration and recovery after CNS injury. In addition to requiring a robust regenerative response from the injured neuron itself, successful axon regeneration is dependent on the coordinated efforts of non-neuronal cells which release extracellular matrix molecules, cytokines, and growth factors that support axon regrowth. The inflammatory response is initiated by axonal disintegration in the distal nerve stump: this causes blood-nerve barrier permeabilization and activates nearby Schwann cells and resident macrophages via receptors sensitive to tissue damage. Denervated Schwann cells respond to injury by shedding myelin, proliferating, phagocytosing debris, and releasing cytokines that recruit blood-borne monocytes/macrophages. Macrophages take over the bulk of phagocytosis within days of PNI, before exiting the nerve by the circulation once remyelination has occurred. The efficacy of the PNS inflammatory response (although transient) stands in stark contrast with that of the CNS, where the response of nearby cells is associated with inhibitory scar formation, quiescence, and degeneration/apoptosis. Rather than efficiently removing debris before resolving the inflammatory response as in other tissues, macrophages infiltrating the CNS exacerbate cell death and damage by releasing toxic pro-inflammatory mediators over an extended period of time. Future research will help determine how to manipulate PNS and CNS inflammatory responses in order to improve tissue repair and functional recovery.

## Introduction

### Nerve injury may have caused World War I

In 1914, Austria's Archduke Ferdinand was assassinated in Sarajevo. Rather than acting with diplomacy, Kaiser Wilhelm II - leader of Germany and Prussia - engaged in warfare with Serbia, ultimately starting World War I. According to historical records ([1,2] but see [3]), the Kaiser's petulant and outspoken demeanour had foundations laid during childbirth: complications during his breech delivery likely caused injury to his brachial plexus nerves, which led to a permanently limp left arm. His mother, Victoria, favoured her healthier children over her flawed eldest son, which created deep-seated insecurities and bitterness in the future Kaiser. Therefore, obstetric brachial plexus injury - and events precipitated by the injury - were instrumental in moulding the Kaiser's perspective and character which ultimately may have started a devastating world war. It is bittersweet irony that many of the most effective treatments for peripheral nerve injury (PNI) were developed during the war: 18% of extremity injuries included trauma to peripheral nerves, allowing physicians to experiment with new therapies. Nerve grafting, which is the current gold standard for PNIs with gaps, was refined during this time [4-7]. Therefore, while nerve injury may have laid the foundation for World War I, treatments for PNIs were vastly improved by innovative surgeons during the war.

Although PNS axons have the capacity to regrow, functional recovery in humans is often incomplete. This is because the regenerative response of the injured neuron and of cells surrounding the injured neuron's axon, cannot maintain an effective growth-promoting response for long periods. Revealing cellular processes and molecular mechanisms that enhance or limit axon regeneration will be instructive for improving clinical outcomes after PNI. In addition, by studying factors that influence PNS axon regeneration, we may discover treatments that improve repair after spinal cord injury (SCI) or brain injury.

In this review, we discuss the initiation of inflammatory cascades by axon degeneration, and the roles of Schwann and immune cells in degeneration and regeneration after PNI (For review on the neuron response to injury, see [8-10]). We then compare the PNI-induced inflammatory response with that elicited by SCI.

### Responses extrinsic to the neuron after nerve injury

**Figure 1:**
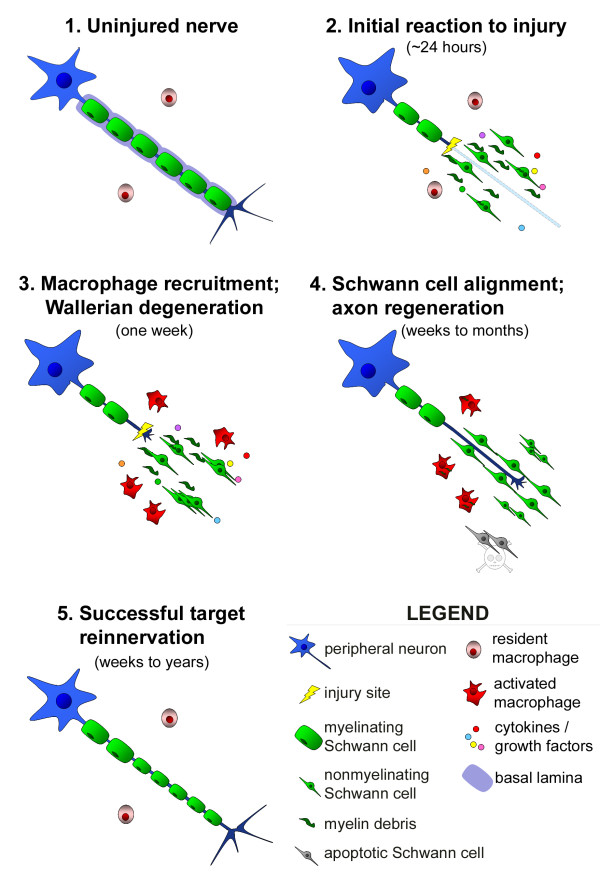
**Progression of Wallerian degeneration and axon regeneration after peripheral nerve injury (PNI)**. A single axon with associated myelinating Schwann cells is shown. Although myelin phagocytosis and degeneration occurs within the basal lamina (purple), the basal lamina is shown only in panel 1 for clarity. **1**. The endoneurium of an uninjured nerve consists of axons, associated Schwann cells (myelinating and nonmyelinating), and resident, inactivated macrophages. **2**. Soon after PNI, denervated myelinating Schwann cells release their myelin. These Schwann cells then proliferate within their basal lamina tubes, produce cytokines/trophic factors, and phagocytose detached debris. In addition, the reaction within the neuron cell body begins: this is characterized by cell soma hypertrophy, displacement of the nucleus to an eccentric position, and dissolution of Nissl bodies. **3**. Wallerian degeneration is well underway within a week of injury. Soluble factors produced by Schwann cells and injured axons activate resident macrophages and lead to recruitment of hematogenous macrophages. The activated macrophages clear myelin and axon debris efficiently, and produce factors that facilitate Schwann cell migration and axon regeneration. **4**. After a lag period, injured axons form a growth cone and begin to regenerate along bands of Büngner formed by Schwann cells. These tubes provide a permissive growth environment and guide extending axons towards potential peripheral targets. Schwann cells that have been chronically denervated (e.g., for a few months) are less supportive of regrowth and are more likely to undergo apoptosis. **5**. If the axon is able to traverse the injury site, and its environment supports its growth along the entire distal stump, then the axon can connect with peripheral targets. Although myelinating Schwann cells do remyelinate the regenerated portion of axon, the myelin is thinner and the nodal length is shorter than in the uninjured portion of axon. See text for references.

Injury elicits a vigorous response from non-neuronal cells in the peripheral nerve, especially in the distal nerve stump (Figure 1). This degenerative process is called Wallerian degeneration after Augustus Volney Waller, who first characterized morphological changes in sectioned frog glossopharyngeal and hypoglossal nerves 160 years ago ([11]; see [12]). The intrinsic degeneration of detached distal axons has been identified as the key event in Wallerian degeneration, triggering a cascade of non-neuronal cellular responses that leads to clearing of inhibitory debris in the peripheral nerve and to the production of an environment that supports axon regrowth for months after injury [13-15].

### Axonal degeneration

Axon degeneration in the distal nerve instigates subsequent degenerative processes after PNI; however, axon degeneration does not begin immediately. Detached axon segments remain intact for days after PNI, and can still transmit action potentials when stimulated [16,17]. The lag between injury and axon degeneration is 24-48 hours in young rats [18,19] and mice [20], whereas it takes several days for primate (including human) axons to degenerate [21,22]. Eventually, axons bead and swell before catastrophic granular disintegration of the cytoskeleton occurs [23,24]. Granular disintegration of the cytoskeleton - the sudden destruction of cytoskeletal elements into fine debris - is completed within an hour [20,25].

Interestingly, mechanisms intrinsic to the detached axon underlie injury-induced degeneration. Calcium entry into the axoplasm from extracellular and intracellular stores is required to initiate the process [26,27]. Calcium influx activates calpain, a protease essential for cytoskeletal degradation and axon degeneration [28,29]. Activation of the axon's ubiquitin-proteasome system has also been implicated in these degenerative processes [30].

The most compelling evidence that supports a pivotal position for axon degeneration in Wallerian degeneration comes from studies involving the slow Wallerian degeneration (Wld^S^) mouse (reviewed by [31]). The Wld^S ^mouse has nerves that degenerate much slower than wild-type nerves [32,33]. Intact axons are found in Wld^S ^distal nerves at 35 days after sciatic nerve transection (20-30% of axons remain), whereas all wild-type axons degenerate by this time [33]. In addition, whereas injured distal nerve segments of wild-type mice can transmit compound action potentials for only 2-3 days upon stimulation, Wld^S ^distal nerves can conduct action potentials for up to 3 weeks [17,32,34]. Axonal granular disintegration of cytoskeleton is significantly delayed in Wld^S ^mice due to intrinsic properties in the axon [33,35-37]. Still, this degenerative process precedes later events including myelin sheath breakdown, macrophage accumulation and axonal regeneration [38]. Therefore, the timing of granular disintegration of the cytoskeleton and axon degeneration defines when subsequent, more extensive degenerative processes begin.

A variety of changes occur in the nerve soon after PNI-induced axon degeneration, including blood-nerve barrier compromise and initiation of cellular changes associated with degeneration. The blood-nerve barrier (and the blood-brain barrier) comprises non-fenestrated endothelial cells connected by tight junctions, and it restricts the movement of proteins, hormones, ions, and toxic substances from blood into neural tissue [39-43]. Although the blood-nerve barrier is often breached at the lesion site after injury, it is not compromised elsewhere along the nerve until axon degeneration begins. At that point, the barrier is partially compromised along the length of the nerve distal to injury for at least four weeks post-injury [44]. Maximal post-transection perineurial permeability, which is double the nerve's normal permeability, occurs within 4-7 days and corresponds with the peak of the acute inflammatory response [45,46]. Increased blood-nerve barrier permeability allows blood-borne factors and cells that will facilitate tissue repair to enter the nerve. Perineurial blood-nerve barrier permeability decreases around 2 weeks after injury, before a second, sustained increase in permeability occurs starting ~4 weeks after transection. This second increase in permeability may reflect changes required to regain homeostasis after Wallerian degeneration [46].

### First responders: The Schwann cell response to peripheral nerve injury

During the three or four weeks following the section, nearly all the nerve sprouts present in the peripheral stump are devoid of nuclei and myelin sheath. But from the fourth week on and sometimes before, fusiform cells with an elongated nucleus apply themselves around the fibres. These cells are produced by the proliferation of the old cell of Schwann.

                                                   - Ramon y Cajal [47], p. 244

Schwann cells, the ensheathing glial cells of the PNS, are crucial for normal nerve function and for nerve repair. Schwann cells constitute 90 percent of nucleated cells within peripheral nerves [48]. They provide trophic support for developing, mature, and regenerating axons. In addition, basal lamina produced by these cells surrounds the Schwann cell and their associated axon(s), and its components support axonal growth (see below).

There are two types of Schwann cells in adult peripheral nerve: myelinating and ensheathing (non-myelinating) Schwann cells [14]. Myelinating Schwann cells form a multi-layered, membranous myelin sheath around large-calibre axons (motor or sensory). These cells associate with one segment of a single axon and are evenly spaced along the length of the axon. Their myelin provides insulation that allows axons to conduct action potentials much more rapidly than they could in the absence of myelination. In contrast, ensheathing Schwann cells, or Remak cells, loosely ensheath multiple small-diameter unmyelinated axons. The ensheathing Schwann cell's cytoplasmic processes segregate and surround axons.

Soon after PNI, Schwann cells in the distal nerve begin to dedifferentiate, a process that is dependent on the ubiquitin-proteasome system [49]. Myelinating Schwann cells associated with detached axons respond to injury, even before axon degeneration occurs, by altering gene expression [50-52]. Within 48 hours of injury, these Schwann cells stop producing myelin proteins [53,54], upregulate regeneration-associated genes (GAP-43, neurotrophic factors and their receptors, neuregulin and its receptors), and begin to proliferate [51,52,55]. Both myelinating and ensheathing Schwann cells divide, reaching peak proliferation around 4 days post-injury. Proliferating Schwann cells are confined to their basal lamina tubes where they align to form bands of Büngner, which provide a supportive substrate and growth factors for regenerating axons [14,56].

Recent studies have shed light on how Schwann cells and immune cells initially sense injury to nearby axons. Toll-like receptors (TLRs), originally defined by their ability to detect microbial pathogens and activate an inflammatory response within cells [57], have since been implicated in recognition of tissue damage through binding of endogenous ligands not normally present in the extracellular milieu (e.g., heat shock proteins [58], mRNA [59], degraded extracellular matrix (ECM) components [60]). Schwann cells express a variety of TLRs - TLR3, TLR4, and TLR7 are constitutively expressed in unstimulated cells - and TLR1 is upregulated after axotomy [61]. The expression pattern of TLRs suggests that Schwann cells perform a sentinel role in the PNS. Indeed, PNI induces TLR-dependent changes in activation of transcription factors, cytokine expression, and progression of Wallerian degeneration and functional recovery. In vitro, adding necrotic neurons, which contain putative TLR ligands, to Schwann cells augments their expression of inflammatory mediators including TNF-α, iNOS, and MCP-1 mRNA; this effect is substantially diminished using Schwann cells from TLR2- or TLR3-deficient mice [62]. Karanth et al. [63] showed that MCP-1 mRNA is induced in cultured Schwann cells by freeze-killed, but not viable nerves in a TLR4-dependent manner, and that 1-10 kDa protein(s) are responsible for this effect. Boivin and colleagues [64] showed that mice deficient in TLR signaling (TLR2-, TLR4-, or MyD88-deficient mice) exhibit reduced pro-inflammatory cytokine expression and macrophage accumulation in distal sciatic nerve, delayed Wallerian degeneration, and impaired functional recovery. Conversely, a single injection of TLR2 or TLR4 ligands expedites myelin clearance and functional recovery. Taken together, these data indicate that endogenous TLR ligands, which are liberated from disintegrating axons, bind TLRs found on Schwann cells and immune cells (e.g., macrophages) leading to activation of inflammatory cascades that may be essential for promoting axon regeneration. In addition to TLRs, emerging data suggest that P2 receptor ligands (e.g., purines) act on Schwann cells to elicit axotomy-induced inflammation (reviewed by [65]).

Schwann cells also play an early role in removing myelin debris, which acts as a barrier to regrowing axons in the distal nerve. After axons degenerate and disappear, Schwann cell myelin sheath partitions longitudinally to form small ovoids [56]. This myelin debris contains molecules that are inhibitory to axonal growth including myelin-associated glycoprotein (MAG) and oligodendrocyte-myelin glycoprotein (OMgp) [66,67]. Schwann cells play an active role in removing myelin debris derived from dying or damaged Schwann cells: they can degrade their own myelin and phagocytose extracellular debris. Schwann cells also express major histocompatibility complex (MHC) class II molecules; however, whether they can act as antigen presenting cells is not clear [68-70]. Denervated Schwann cells are the major phagocytic cells for the first 5 days after injury [71].

The initial degradation of myelin after PNI is dependent on activation of the phospholipase-A_2 _(PLA_2_) family of enzymes (see [72]). Cytosolic and secreted forms of PLA_2 _hydrolyze the phospholipid phosphatidylcholine into lysophosphatidylcholine (which elicits myelin breakdown) and arachadonic acid (which can be metabolized into pro-inflammatory eicosanoids). In other cell types, PLA_2 _expression and activation is increased by cytokines (e.g., TNF-α, IL-1β; [73,74]); thus, these inflammatory mediators may also control PLA_2 _activation in Schwann cells after PNI. Interestingly, cytosolic and secreted forms of PLA_2 _are upregulated in Schwann cells and macrophages within hours of injury and remain elevated for 2 weeks [74]. This time course correlates with Wallerian degeneration after PNI, and the return to basal PLA_2 _levels 3 weeks post-PNI is associated with axon regeneration and remyelination. De et al. [74] also found that blocking PLA_2 _expression/activity in sciatic nerves distal to injury significantly prolonged clearance times for degenerating myelin and axons. Therefore, PLA_2 _activity is required for the initiation of myelin breakdown and for progression of Wallerian degeneration after PNI.

In addition to proliferating and phagocytosing debris after PNI, Schwann cells in the distal nerve stump secrete trophic factors that promote axon growth along with cytokines and chemokines that recruit immune cells into the injured nerve. Within a week of injury, recruited immune cells - especially macrophages - assume a primary role in debris removal and growth factor production in the next stage of Wallerian degeneration. After sciatic nerve transection in mice, Schwann cells in the distal nerve synthesize the pro-inflammatory cytokines TNF-α and interleukin (IL)-1α within 5 h, whereas IL-1β production is delayed until ~24 h [75]. Likewise, expression of IL-6 and LIF mRNA is increased within 3 hours of PNI [76-79]; these factors are produced by Schwann cells and are required for immune cell chemotaxis [80]. In fact, IL-6 treatment of Schwann cells increases expression of LIF and MCP-1 (monocyte chemoattractant protein-1, a.k.a. CCL2) mRNA, and LIF treatment of Schwann cells increases MCP-1 mRNA [80]. TNF-α also elicits production of MCP-1 production [81] and matrix metalloproteinase (MMP)-9 [82]; both are necessary for axotomy-induced macrophage accumulation. Collectively, these data indicate that activated Schwann cells initiate cytokine/chemokine cascades that amplify and fine-tune the inflammatory response after PNI.

Schwann cells promote axon regeneration by secreting ECM molecules and trophic factors [83]. Laminins, which are the second-most prevalent ECM component in the PNS (after collagen), support robust axon growth. Two of the 15 laminin trimers are expressed in intact peripheral nerves (laminin 2 - α2β1γ1, laminin 8 - α4β1γ1) [84], and their expression increases after PNI [85]. Conditional knockout of the γ1 laminin subunit in Schwann cells, which practically eliminates expression of functional laminin in the PNS, alters Schwann cell physiology (e.g., errors in differentiation and axon ensheathment) and strongly inhibits axon growth after sciatic nerve lesion [86]. Therefore, laminins are indispensible for maintenance and repair of injured peripheral nerves. Laminins act directly on neurons and Schwann cells by binding specific receptors (integrins and dystroglycans) that enhance adhesion and provide a molecular link between the ECM and the actin cytoskeleton, thereby eliciting neurite outgrowth and myelination (e.g., [87-89]).

Schwann cells also secrete different trophic factors that support neuron survival and growth. The pro-inflammatory cytokine IL-6, which is elevated in both neurons and non-neuronal cells after PNI [78,90], signals via its receptor to increase expression of regeneration associated genes (RAGs) in neurons and promote neurite growth [91,92]. Neurotrophic factors such as nerve growth factor (NGF) are elevated by nerve injury and may promote axon regrowth [93-97], although these proteins are not necessary for peripheral axon regeneration in all injury models [98,99].

Although Schwann cells release myriad growth factors and cytokines, in some cases these cells limit the availability of secreted growth-promoting factors by binding these proteins themselves. Using a co-culture system that included Schwann cells and dorsal root ganglion (DRG) neurons, the Ramer laboratory showed that expression of the neurotrophic factor-binding receptor p75^NTR ^by Schwann cells restricts the ability of axons to grow in response to neurotrophic factors [97]. Wild-type neurons cultured on p75^NTR^-null Schwann cells extended more neurites than those cultured on wild-type Schwann cells. This effect was abolished by treatment with Trk-Fc (a soluble Trk receptor), suggesting that sequestration of neurotrophic factors by Schwann cell p75^NTR ^limits the regenerative potential of injured peripheral axons. *In vivo*, Scott et al. [97] studied regeneration of axons from DRG neurons into a peripheral nerve graft in the dorsal column of the spinal cord. More wild-type axons regenerated into p75^NTR-/- ^(versus wild-type) grafts. Axon regeneration after dorsal root injury was also examined. In this model, injured peripheral axons normally cannot regenerate beyond the PNS-CNS border (i.e., the dorsal root entry zone). Interestingly, axons in p75^NTR-/- ^mice regenerated into the CNS after dorsal root injury, and this process was inhibited by Trk-Fc infusion. Therefore, although Schwann cells release growth factors, they also bind some of these proteins thereby titrating their ability to promote axon growth. How Schwann cell physiology is affected by the binding of neurotrophic factors to cell surface receptors (including p75^NTR^) remains to be elucidated.

Other factors enhance nerve repair indirectly. For example, neuron-derived neuregulin promotes Schwann cell migration and aids in recovery [100]. Compared with wild-type axons, injured transgenic mouse axons lacking neuregulin-1 regenerate more slowly, display aberrant terminal sprouting at the neuromuscular junction and are hypomyelinated [101].

Although Schwann cells in the distal nerve initially mount an effective response that promotes axon regeneration, their ability to survive and support axon growth declines within eight weeks of denervation [102,103]. This time-dependent loss of support in the distal nerve is one factor that limits successful long-distance peripheral axon regeneration. Many denervated Schwann cells die by apoptosis within months after injury and leave behind their basal lamina, which is eventually degraded and removed [15,55,104,105]. Moreover, those Schwann cells that persist in the chronically injured nerve begin to atrophy and fail to support axon growth [15,104-106]. Interestingly, chronically denervated Schwann cells can be reactivated by treatment with TGF-β, a cytokine that is released by proliferating Schwann cells and macrophages. Reactivated Schwann cells support axon regeneration [105]. In summary, denervated Schwann cells promote nerve repair by proliferating, secreting trophic factors and cytokines, and phagocytosing myelin debris; however, this support diminishes after 1-2 months of chronic denervation.

### Specialized back-up: The immune cell response to peripheral nerve injury

The typical immune cell response to tissue injury and infection is largely conserved in the pathological PNS: many phagocytic neutrophils and macrophages arrive within hours or days post-injury, whereas lymphocyte accumulation in the distal injured nerve segment is delayed by a week or more.

Neutrophils (polymorphonuclear granulocytes), the first inflammatory leukocytes to invade injured tissue from the circulation, phagocytose debris and modulate recruitment and activation of other leukocytes (mainly monocytes) during Wallerian degeneration [107]. Although neutrophils are sparsely distributed in the uninjured rat nerve, their density in the area immediately around the injury site increases substantially within 8 hours, and peaks at 24 hours (only a few neutrophils infiltrate more distal areas of the distal stump) [108]. These cells have a high rate of turnover; after entering tissue, neutrophils briefly phagocytose debris before undergoing apoptosis [109]. It is not known how neutrophils affect regeneration of injured peripheral axons.

**Figure 2:**
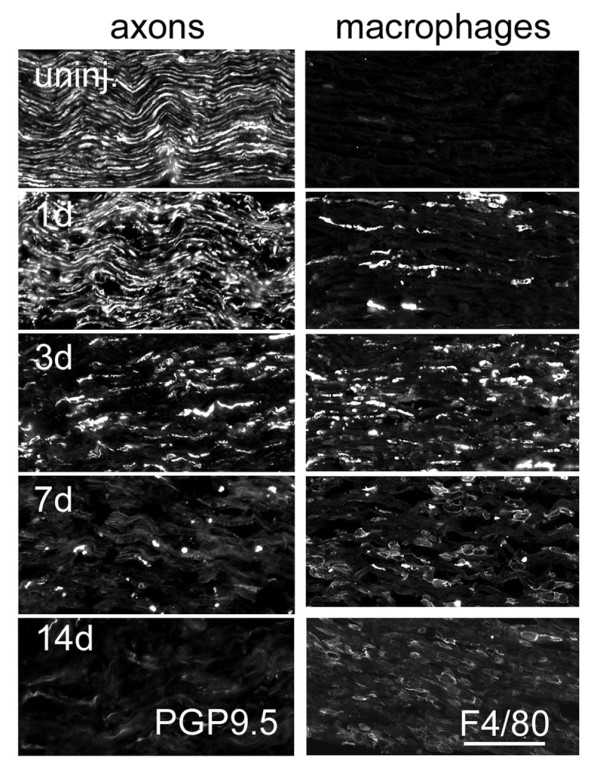
**Progression of axon degeneration (left panels) and macrophage accumulation (right panels) in mouse distal nerves after tight sciatic nerve ligation**. Sciatic nerves from 129P3/J mice were harvested at the indicated timepoints post-injury. After sectioning nerves longitudinally, we used immunohistochemistry to visualize axons (PGP9.5) and macrophages (F4/80). Axons degenerate progressively, with early discontinuities visible within 1 day of injury, extensive degeneration at 3 days, and nearly complete degeneration within 7 days (axon regeneration is precluded by tight ligation of the sciatic nerve). Few macrophages reside within the uninjured (uninj.) nerve. Macrophage accumulation, which includes resident macrophage proliferation and hematogenous macrophage infiltration, begins by 1 day after injury and peaks between 3 and 7 days post-axotomy. Note the change in macrophage morphology and F4/80 immunoreactivity between 3 and 7 days: compact, elongated F4/80-positive cells predominate at 3 days, whereas most macrophages are large and amoeboid at 7 days post-axotomy. This switch reflects the phagocytosis of large amounts of debris by macrophages between those timepoints. Scale bar, 200 μm.

Macrophages are another major immune cell population that respond to PNI (Figure 2). Importantly, they remove myelin debris during later phases of Wallerian degeneration. Endoneurial macrophages account for 2-9% of nucleated cells within the uninjured peripheral nerve [110-112]. These resident macrophages express MHC molecules and complement receptor 3; these surface molecules endow macrophages with antigen presenting and surveillance functions, respectively [14,110,113]. Using sciatic nerve explants, resident macrophages were found to respond to PNI by proliferating and by phagocytosing myelin [67,114]. Endoneurial macrophage proliferation, activation, and phagocytic activity commence within 2 days post-axotomy, before blood-derived monocytes infiltrate the injury site [115].

Although proliferating endoneurial macrophages contribute significantly to the early stages of Wallerian degeneration, large numbers of circulating monocytes accumulate within the injured nerve by four days post-injury [110]. Monocytes, which differentiate into macrophages in the tissue, are recruited from the blood by Schwann cell- and macrophage-derived cytokines and chemokines, such as LIF, MCP-1 and TNF-α ([80,116]; see above). Local cues direct macrophages to distinct areas of damage: after injury to a subset of axons within a nerve, macrophages accumulate preferentially around the injured fibres [56]. Hematogenous macrophage accumulation in the injured nerve is also enhanced by serum components such as antibodies and complement, as macrophage recruitment is delayed in mice deficient in B lymphocytes (which cannot produce antibodies) [117] or complement [118]. Breakdown of the distal nerve's blood-nerve barrier within 48 hours of injury allows influx of these serum components [119], which then facilitate macrophage recruitment and label or "opsonize" debris to facilitate phagocytosis [120,121].

One factor implicated in PNI-induced macrophage accumulation is the 14.5 kDa protein galectin-1. Galectin-1 is expressed by macrophages, Schwann cells and neurons within peripheral nerves, and its expression is elevated within three days of axotomy. We found that injury-induced macrophage accumulation is delayed and diminished in galectin-1 null mutant mice [122]. Conversely, injection of the oxidized form of galectin-1 into intact wild-type nerve augmented the accumulation of macrophages similar to that observed after injection of the potent inflammogen, zymosan. In fact, galectin-1 is chemotactic for monocytes, but not macrophages [123]. Horie and colleagues [124] showed that oxidized galectin-1 binds to an unidentified receptor on macrophages, resulting in secretion of a factor that promotes Schwann cell migration and axon regrowth. The ability of galectin-1 to enhance peripheral axon regeneration and functional recovery is likely mediated by its diverse effects on macrophages [125-127].

Hematogenous macrophages are essential for effective myelin phagocytosis [128,129] and produce cytokines that activate Schwann cells (e.g., IL-1 [130]) and trophic factors that aid axon regeneration (e.g., NGF [93,111,131]). Neurites from DRG explants, which normally grow minimally on uninjured nerve cryosections, become stabilized and grow more robustly when nerve sections are treated with macrophage-conditioned medium or are derived from pre-degenerated nerves [132], supporting a role for macrophages in PNS axon regrowth. Macrophages also re-model the distal nerve's ECM in preparation for regrowing axons [130].

**Figure 3:**
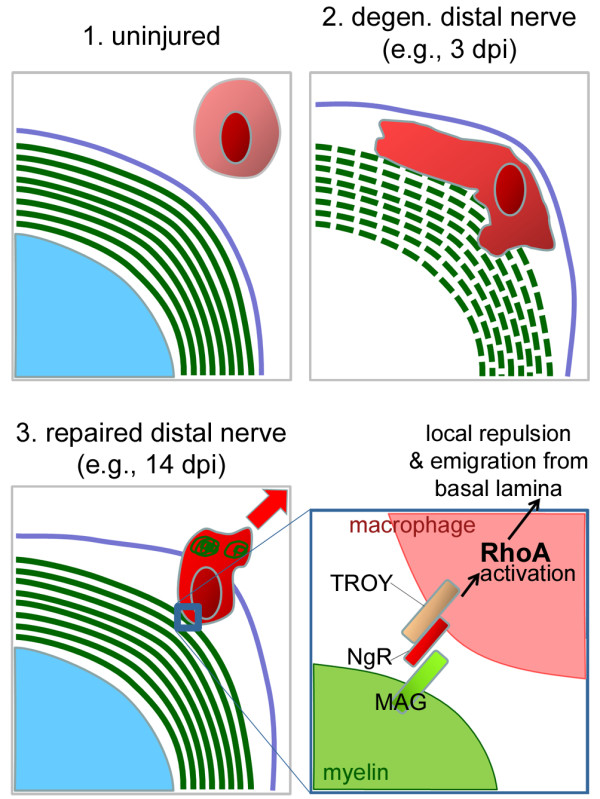
**After injury and regeneration, proteins in newly-formed myelin contribute to resolution of the inflammatory response by facilitating macrophage exit from the basal lamina**. A single axon (turquoise) with myelin (green) and basal lamina (purple) is shown in cross-section. **1**. In the uninjured peripheral nerve, the myelinated axon is surrounded by many tight wraps of myelin; this unit is covered by the basal lamina. Resident macrophages (pink) perform a surveillance role and are present outside of the basal lamina. **2**. After peripheral nerve injury, the axon degenerates and myelin break down begins. Activated resident and hematogenous macrophages accumulate and penetrate the basal lamina, where they phagocytose myelin and axon debris. Because the debris can physically prevent regeneration and also contains inhibitors to axon growth, this macrophage-mediated phagocytosis is a crucial step in nerve repair. **3**. After debris phagocytosis, axon regeneration, and remyelination, macrophages are no longer useful within the basal lamina. Proteins on the surface of newly-formed myelin signal debris-laden macrophages to emigrate from the basal lamina. **Inset (fourth panel): **myelin-associated glycoprotein (MAG), present on myelin membranes, interacts with the receptor NgR and its signaling partner TROY on macrophage membranes. Engagement of this receptor complex in the trailing edge of macrophages leads to local activation of the small GTPase RhoA, which signals for local repulsion and movement away from the source of activation (myelin). Ultimately, this causes macrophage exit from the basal lamina once remyelination has occurred.

Excess macrophages remain in the nerve for days to months, after which they either emigrate to lymphatic organs via the circulation or die by apoptosis [72,133]. David's group has studied macrophage emigration in detail [72] (Figure 3). To phagocytose degenerating myelin, macrophages must penetrate Schwann cell basal lamina tubes; therefore, in order to leave, the cells must receive a signal and emigrate through the basal lamina to nearby vessels. NgR1 and 2 - which are widely known as receptors that bind myelin-associated inhibitory proteins - are expressed on macrophages, and a higher proportion of cells express these proteins at seven days post-axotomy, when many are phagocytic [134]. Upon contact with myelin surrounding remyelinated axons, these NgR-expressing macrophages are repelled (via RhoA activation) and exit basal lamina tubes, eventually re-entering the circulation (see also [135]).

The last immune cells to arrive in the injured nerve are T lymphocytes. These cells infiltrate the injured sciatic nerve by 3 days after chronic constriction injury then reach peak numbers 14-28 days after injury [136]. T lymphocytes shape the later phase of the immune response by producing pro- or anti-inflammatory cytokines that support cellular and humoral immunity [137]. Whereas pro-inflammatory cytokines (e.g., TNF-α, IFN-γ) secreted by Type 1 helper T (Th1) cells activate nearby macrophages, neutrophils and natural killer cells, anti-inflammatory cytokines (e.g., IL-4, IL-10) released by Type 2 helper T (Th2) cells inhibit various macrophage functions and suppress/regulate pro-inflammatory cascades [138,139]. Using transgenic mice, Beahrs et al. [140] showed that a Th2 response supports survival of a small subset of facial motoneurons after axotomy, whereas both Th1 and Th2 cells are necessary to promote typical axon regeneration.

Therefore, PNI initiates an inflammatory response that is widespread, involves multiple cell types, and lasts for months. Whereas Schwann cells mediate myelin clearance in early stages of Wallerian degeneration, resident endoneurial and hematogenous macrophages play a crucial role in debris removal and nerve repair beginning within a week of PNI. For instance, conditional depletion of CD11b-positive macrophages starting 12 h prior to sciatic nerve crush in transgenic mice resulted in reduced myelin debris clearance, loss of neurotrophin synthesis, and decreased axon regeneration and functional recovery [141]. In addition, Lu and Richardson [142] showed that macrophage activation around the cell body of injured dorsal root ganglion neurons can enhance regeneration of their axons, suggesting that injury-induced macrophage activation in areas far from primary injury (but still nearby affected cells) can also improve nerve repair. Therefore, it is widely believed that peripheral axon regeneration is dependent on a rapid and efficient macrophage response.

### Responses extrinsic to the neuron after CNS injury

In contrast with the efficient response coordinated within injured peripheral nerves, non-neuronal responses to CNS injury contribute to regenerative failure. CNS injury elicits changes in blood-brain barrier permeability, in cells' activation states, in the cellular composition of the CNS, and in the extracellular milieu.

Whereas the PNI-induced increase in blood-nerve barrier permeability occurs over a large distance and is long-lasting, alterations in the blood-brain barrier are smaller in scale following CNS injury. After spinal cord contusion, the permeability of the blood-brain barrier increases at and around the injury site for about three weeks [39,143]. Even though granular disintegration of the axon cytoskeleton occurs relatively soon after CNS injury (as in the PNS), the blood-brain barrier is not fully compromised in areas associated with degenerating CNS tracts. Therefore, the maintenance of the blood-brain barrier that covers disconnected CNS tracts could be a factor that underlies protracted Wallerian degeneration - and poor axon regeneration - in the pathological spinal cord and brain. However, given the unique environment of the normally immune-privileged CNS and the largely detrimental effects of CNS macrophages (see below), increasing blood-brain barrier permeability to enhance debris clearance is not likely to be an effective option for improving regeneration or recovery.

The growth-supportive phenotype of denervated Schwann cells contrasts starkly with the response of endogenous CNS glia to injury [144,145]. Oligodendrocytes, the myelinating cells of the CNS, respond to axonal injury by either undergoing apoptosis or entering a quiescent state [146-148]. Compared to Schwann cells, oligodendrocytes are more sensitive to axonal injury, provide minimal growth support, and have little phagocytic activity [149-152]. Astrocytes respond to injury by proliferating and undergoing hypertrophy. Together, these physical changes help to create a "glial scar" that effectively encloses sites of CNS injury and helps to modulate inflammatory cascades. Activated astrocytes also produce factors (e.g., CSPGs) that inhibit axon growth [153,154].

Compared to that in the PNS, inflammation in the CNS is significantly more cytotoxic due in part to a protracted pro-inflammatory macrophage response. Macrophages are the primary effectors of inflammation after SCI and are attracted to the spinal cord in large numbers soon after injury. The phenotype of the acute macrophage response to SCI can be categorized as pro-inflammatory or "M1" [155,156]: while M1 cells sterilize wounds and promote tissue repair, they also release pro-inflammatory cytokines, proteolytic enzymes and free radicals. In non-CNS tissue, the macrophage response is then re-programmed into an anti-inflammatory "M2" phenotype [156,157] (although M1-M2 macrophage polarization in the injured PNS remains to be characterized). M2 cells release anti-inflammatory cytokines and protect surrounding cells, promoting angiogenesis and healing at later stages of recovery [158]. Popovich's group showed that this natural progression from M1 to M2 macrophages does not occur after SCI [155]. Instead, M1 macrophages dominate the lesion site indefinitely after SCI. Medium from M1 macrophages promotes neuritic outgrowth from cultured neurons but is also neurotoxic. In contrast, M2 macrophage-conditioned medium elicits more efficient and longer neurite growth than does M1 medium, without killing neurons.

The prevalence of M1 macrophages, and their conflicting effects on growth and survival of cultured neurons, may explain why CNS macrophages provoke both secondary damage and repair after SCI [159]. Given that the harmful effects of macrophages seem to predominate, altering the phenotype of the macrophage response (e.g., promoting M2 cells) may be an effective strategy for improving repair after SCI.

In addition to the neurotoxic effects of neuroinflammation in the injured CNS, the efficiency with which resident or recruited macrophages remove debris from the CNS is reduced relative to the PNS. Indeed, myelin debris can be found in the degenerating human corticospinal tract years after injury [160,161]. Nevertheless, enhancing phagocyte efficiency and accelerating the clearance of putative inhibitors of axon growth after SCI may not be sufficient to promote CNS axon regeneration: systemic injection of the inflammatory agent lipopolysaccharide expedited myelin clearance modestly after SCI, but failed to promote regeneration of sensory axons in the dorsal column [162]. Therefore, the limited phagocytic and growth-promoting response of macrophages, microglia and oligodendrocytes may underlie the delayed removal of inhibitory debris during CNS Wallerian degeneration.

The composition of CNS ECM is also very different from that in the PNS. CNS ECM includes the massive glycosaminoglycan hyaluronan and the glycoproteins tenascin-C and thrombospondin [163]. CNS ECM lacks significant amounts of collagen, laminin, and fibronectin, which contribute to the structure and strength of other tissues [164]. In contrast with its wide distribution in the PNS, laminin is confined to the pial and vascular basal lamina in the CNS [163]. In addition, oligodendrocytes do not have an associated basal lamina [87], which is a key contributor to PNS axon regeneration. Finally, after CNS injury, the dense glial scar and ECM network that forms at the injury site acts as a physical and molecular barrier to axon regeneration [165]. Due to protracted Wallerian degeneration, myelin-associated inhibitory factors may impact injured CNS axons. In addition to MAG and OMgp (which are also present in the PNS), degenerating CNS myelin contains the outgrowth inhibitor NogoA [166,167]. These inhibitors linger at and around the injury site, where they may contribute to regenerative failure (but see [168-170]).

In summary, changes in the environment of injured CNS neurons and axons effectively prohibit axon regeneration. Endogenous CNS cells contribute to the hostile environment: astrocytes proliferate and release inhibitory factors, oligodendrocytes atrophy and release myelin-associated inhibitors, and microglia operate as sub-optimal phagocytes and possibly as neurotoxic effector cells.

## Conclusions

In summary, every sectioned nerve regenerates its axons by means of sprouts from the central stump which, as Tello proved, cross the scar and assail the peripheral stump to reach the external sensory and muscular terminations. Arriving at their destination, attracted no doubt by some substance (or physical influence as yet unknown) arising from the nuclei of the terminal apparatus, the destroyed motor arborization moulds itself anew.

                                                   - Ramon y Cajal [47], p. 92

Here, Ramon y Cajal refers to the remarkable regenerative capacity of peripheral nerves. It is now abundantly clear that successful peripheral axon regeneration is associated with a rapid and efficient inflammatory response that is terminated in due course. Schwann cells and macrophages, the major cellular constituents in the distal nerve stump undergoing Wallerian degeneration, communicate via cytokine networks and exhibit exquisite control over phagocytosis and growth factor release in the distal nerve, setting the stage for axon regeneration. However, these cells lose their ability to promote axon regrowth in the chronically denervated distal stump, precluding long distance axon regeneration in humans. In addition, they cannot support growth over nerve gaps caused by mechanical injury. After CNS injury, the cellular and molecular cascades associated with Wallerian degeneration are inadequate for removing presumed inhibitory myelin debris, and most macrophages that occupy injured axonal tracts have a neurotoxic phenotype and prevent effective long distance axon growth. Therefore, discovering treatments that manipulate inflammatory cells in a way that protects nearby cells and enhances axon growth would likely lead to enhanced recovery of patients after PNI or SCI.

## List of abbreviations

CNS: central nervous system; DRG: dorsal root ganglion; ECM: extracellular matrix; GAP-43: 43 kDA growth-associated protein; IFN-γ: interferon-γ; IL-: interleukin (e.g., IL-6); LIF: leukemia inhibitory factor; MAG: myelin-associated glycoprotein; MCP-1: monocyte chemoattractant protein-1 (a.k.a. CCL-2); MMP: matrix metalloproteinase; NGF: nerve growth factor; NgR: Nogo receptor; OMgp: oligodendrocyte-myelin glycoprotein; p75^NTR^: p75 neurotrophin receptor; PLA_2_: phospholipase-A_2_; PNI: peripheral nerve injury; PNS: peripheral nervous system; RAG: regeneration-associated gene; SCI: spinal cord injury; Th1 (or 2): Type 1 (or 2) helper T cells; TLR: toll-like receptor; TNF-α: tumour necrosis factor-α; Trk: tropomyosin-related kinase; Wld^S^: slow Wallerian degeneration mouse.

## Competing interests

The authors declare that they have no competing interests.

## Authors' contributions

ADG conceived and drafted the manuscript. PGP and MSR helped write and revise the manuscript. All authors read and approved the final paper.
